# Metabolic syndrome for sub-Saharan Africans diabetes with peripheral arterial disease: a case-control study

**DOI:** 10.1186/1756-0500-7-104

**Published:** 2014-02-24

**Authors:** Jean Joel R Bigna, Jean Bahebeck, Eugène Sobngwi, Jean Claude Mbanya

**Affiliations:** 1 Faculty of Medicine and Biomedical Sciences, University of Yaoundé 1, P.O. Box 1364, Yaoundé, Cameroon; 2 Goulfey District Hospital, Goulfey, Cameroon; 3 Orthopedic Surgery and Traumatology Department, Yaoundé Central Hospital, P.O. Box 5555, Yaoundé, Cameroon; 4 National Centre of Obesity, Yaoundé Central Hospital, P.O. Box 5555, Yaoundé, Cameroon

**Keywords:** Metabolic syndrome, Peripheral arterial disease, Diabetic foot, Africa, Low-income country, Endocrine disorders

## Abstract

**Background:**

Currently, there is no value for the definition of abdominal obesity by measuring waist circumference in the Sub-Saharan Africa. Several definitions of metabolic syndrome (MS) have disparities concerning use of waist circumference, including International Diabetes Federation (IDF), American Heart Association/National Heart, Lung and Blood Institute (AHA/NHLBI) and National Cholesterol Education Program-Adult Treatment Panel III (NCEP-ATPIII) definitions. The aim of the study was to determine what value of waist circumference should be used and whether to use it as obligatory criterion in the metabolic syndrome in case of peripheral arterial disease (PAD).

**Methods:**

We conducted a case–control study in Cameroon. We included patients with diabetic foot and type 2 diabetes and excluded those with an Ankle Brachial Index (ABI) >?1.3. Cases were defined as patients with ABI?≤?0.9 and controls with ABI >?0.9. The significant p value was < 0.05 and odds ratio (OR) with 95% confidence interval was used to measured risk for have PAD with MS.

**Results:**

We included 19 cases and 48 controls. The risk for having PAD with MS are for the IDF: OR = 4.7 (1.4-15.1), p = 0.008, for the AHA / NHLBI: OR = 5.8 (1.5-22.5), p = 0.007, for the NCEP-ATPIII: OR = 1.8 (0.6-5.6), p = 0.286.

**Conclusion:**

Abdominal obesity should be defined according to the recommendations of the IDF and AHA / NHLBI and should not be an obligatory criterion in the definition of MS for research risk to have PAD on sub-Saharan Africa.

## Background

Africa has diabetes prevalence which is the lowest in the world, but with the rate of undiagnosed patients which is highest [1], one of the important risk factors for type 2 diabetes and cardiovascular risk is metabolic syndrome (MS) [2,3]. A disparity exists between the prevalence of MS in the same population whether subjects with diabetes are included or not. This disparity is due to the existence of several definitions of various medical societies including: the International Diabetes Federation (IDF) [2,4], the National Cholesterol Education Program-Adult Treatment Panel III (NCEP-ATP III) [5,6], the American Heart Association / National Heart, Lung and Blood Institute (AHA / NHLBI) [7]. The diabetic foot is found in 13% patients with type 2 diabetes in Cameroon [8]. In the same study, the prevalence of MS in the population of colored population is 60.6% according to the IDF, 55.4% according to the NCEP-ATP III [9]. In the population of patients with type 2 diabetes in Cameroon, the prevalence is 71.7% according to the IDF criteria and 60.4% according to the NCEP-ATPIII [10]. In Africa since the 2000s, the prevalence of PAD in diabetics varies from 12.5% to 78.7% depending on the study [11]. The relationship between MS and atherogenic risk has already been established by several studies [2,3,7,12]. In the criteria of JIS, it is recommended to use IDF criteria for waist circumference in non-European [3]. But in IDF criteria, there is no data for Sub-Saharan African concerning waist circumference [2]. With the disparity between the various definitions and the lack of information concerning waist circumference for sub-Saharan Africans, the present study aim to orientate towards the definition of waist circumference and MS which are best to use to evaluate risk for have PAD in diabetic foot.

## Methods

### Study design

It was a case–control study conducted in 2012. The study was approved by the administrative authorities of the Yaoundé Central Hospital and the National Ethics Committee of Cameroon. Data were collected after written consent of each patient.

### Setting

The study was conducted at the National Obesity Centre in the Yaoundé Central Hospital, Cameroon. It is a tertiary health care setting.

### Participants

We included in the study all patients with a diabetic foot disease and being diabetic type 2. We excluded patients refusing to participate in the study and patients with an ABI >?1.3 because it was uninterpretable [13,14]. In the case group, we included patients with PAD and in the control group patients without PAD.

### Data

Age, sex and duration of diabetes were collected. The diagnosis of PAD was done using a handheld Doppler and 8 MHz probe. The diagnosis of PAD was based on an ABI ≤ 0.9. The ABI was the ratio of the highest systolic pressure of the ankles to the highest systolic pressure of the arms [13]. The ABI has a level of specificity of 88.5% and sensitivity of 70.6% in type 2 diabetes patients with diabetic foot disease [15]. The sensitivity and specificity are good to allow the usefulness of this test. It is non-invasive method most recommended in search of PAD in the diabetic foot [13].

**Figure 1:**
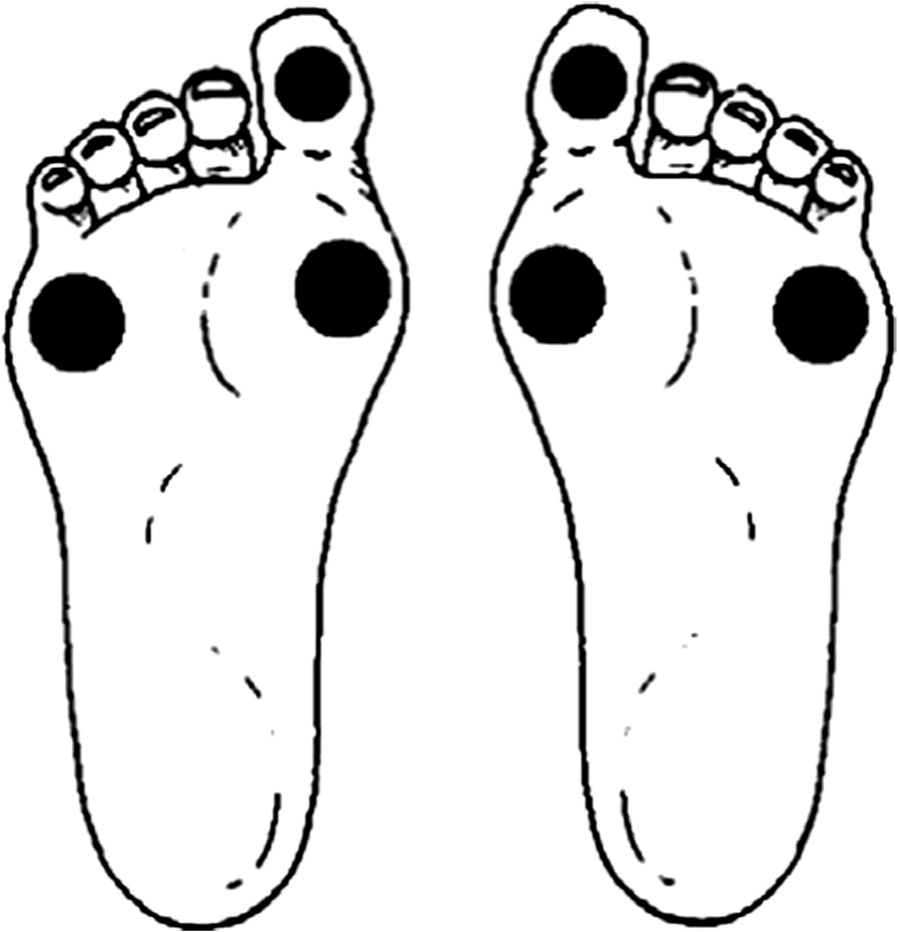
Place of investigation of peripheral neuropathy using 10 g monofilament.

We have collected the level of Total Cholesterol (TC), High Density Lipoprotein (HDL), Low Density Lipoprotein (LDL) and triglycerides (TG) when we received firstly patients. No patient had a treatment for dyslipidemia in the measurement of lipid levels (at the introduction in study). The waist circumference was measured according to WHO recommendations [12]. Peripheral neuropathy was investigated using 10 g monofilament. The filament was applied on sole of the foot at places as indicated in Figure 1. The pressure was made perpendicularly. The diagnosis of sensitive neuropathy was done when two of the three regions were insensitive. We researched impalpable pulses: anterior and dorsal pedal pulses, and popliteal pulses.

Concerning the definitions of MS, we used three different definitions of the following medical societies: IDF, AHA / NHLBI and the NCEP-ATP III. The five elements that constitute the MS are identical for these three definitions: central obesity, increased triglycerides (TG) levels, the blood pressure, fasting plasma glucose and lower high density lipoproteins (HDL). The entry criterion for the definition of the IDF is central obesity with two of the following four elements: TG levels?≥?1.7 mmol/L or specific treatment for abnormal levels of TG, HDL <1.03 mmol/L in men and <1.29 mmol/L in women or specific treatment for abnormal levels of HDL, systolic blood pressure?≥?130 mmHg or diastolic blood pressure?≥?85 mmHg or previous diagnosis of hypertension, fasting plasma glucose?≥?5.6 mmol/L or prior diagnosis of type 2 diabetes [2]. For the last element (type 2 diabetes), all patients in the study had it, it remained to find the other four. For defining central obesity, in the absence of specific data for sub-Saharan African, we used data from the European population for men and?≥?94 cm for women?≥?80 cm [2]. The definition of the NCEP ATPIII-differs from the IDF on three components: central obesity is not an obligatory criterion, the definition of central obesity is made for a waist circumference >?102 cm in men and >?88 cm in women without taking account of ethnic origin, increased fasting plasma glucose is set to blood glucose levels?≥?6.1 mmol/L [6,16]. The definition of the AHA / NHLBI differs from the IDF on a single element: central obesity is not an entry criterion [3]. The most recent, the joint interim statement definition was presented in Table 1[3].

**Table 1:** **Joint interim statement definition of metabolic syndrome**[3]

**Measure**	**Categorical cut points**
Elevated waist circumference*	Population- and country-specific definitions
Elevated triglycerides (drug treatment for elevated triglycerides is an alternate indicator)†	≥ 150 mg/dL (1.7 mmol/L)
Reduced HDL-C (drug treatment for reduced HDL-C is an alternate indicator)†	< 40 mg/dL (1.0 mmol/L) in males
	< 50 mg/dL (1.3 mmol/L) in females
Elevated blood pressure (antihypertensive drug treatment in a patient with a history of hypertension is an alternate indicator)	Systolic?≥?130 and/or diastolic?≥?85 mm Hg
Elevated fasting glucose‡ (drug treatment of elevated glucose is an alternate indicator)	≥ 100 mg/dL

HDL-C = High Density Lipoprotein.

*It is recommended that the IDF cut points be used for non-Europeans and either the IDF or AHA/NHLBI cut points used for people of European origin until more data are available.

†The most commonly used drugs for elevated triglycerides and reduced HDL-C are fibrates and nicotinic acid. A patient taking 1 of these drugs can be presumed to have high triglycerides and low HDL-C. High-dose -3 fatty acids presumes high triglycerides.

‡Most patients with type 2 diabetes mellitus will have the metabolic syndrome by the proposed criteria.

### Statistical analysis

The sample size of each group was calculated using the WinPepi [17]. The ratio in Sub-Saharan Africa of diabetic patients without PAD/with PAD varies from 2.6 to 4.5 using a Doppler [18]. We used 2.6 for the ratio between population sizes of each group because it approaches unity. We assume that the prevalence of metabolic syndrome was 30% in the population of patients without PAD and 70% in the population with PAD. The required size was 16 cases and 42 controls for a supposed power of 80% and p-value of 0.05. Data was coded, entered, and analyzed using the Statistical Package for Social Science (SPSS) version 19 for Windows (SPSS, Chicago, Illinois, USA). Continue variables were expressed as mean (standard deviation) and compared using t-test of Student. Categorical variables were expressed as frequencies (percentages) and compared using the two-tailed Chi-square test or its equivalents. A p-value less than 0.05 was considered statistically significant. Elements of MS were included in the univariate analysis as factors of PAD. All elements of the MS were then incorporated into a multivariate logistic analysis even without significance in the univariate analysis. Two logistic analyzes were conducted: one with central obesity according to the recommendations of the IDF and AHA / NHLBI and other elements of MS, and the other with android obesity as recommended by the NCEP-ATPIII and other elements of the MS.

## Results

### General features

We included 19 subjects in the group of patients with PAD and 48 without PAD. A statistically significant difference was found between patients with and without PAD concerning waist circumference in men and women, HDL and LDL levels in all subjects and among women, TG level in all subjects and among men, and pulse abnormality (Table 2).

**Table 2:** General characteristics of the study population

**Variables**	**PAD (+)**	**PAD (-)**	***p***
	**n = 19**	**n = 48**	
Ankle brachial index (SD)	.75 (.14)	1.09 (.11)	< .001
Age, years (SD)	49.3 (7.2)	50.8 (12.3)	.53
Male, n (%)	11 (57.9)	19 (39.6)	.17
Duration of diabetes, years (SD)	6.9 (3.1)	7.6 (4.6)	.55
Waist circumference, cm (SD)			
- Male	101.2 (9.7)	89.9 (7.1)	.001
- Female	94.6 (5.0)	91.2 (11.6)	.044
Impalpable pulses, n (%)	15 (78.9)	4 (8.3)	< .001
Sensitive peripheral neuropathy, n (%)	16 (84.2)	37 (77.1)	.74
Infection, n (%)	18 (94.7)	47 (97.9)	.49
HbA1c, mmol/mol (SD)	92.4 (9.3)	84.7 (7.1)	.39
Cholesterol, mmol/L (SD)			
- Total Cholesterol	5.65 (1.63)	5.37 (1.64)	.54
• Male	6.63 (1.25)	5.69 (1.50)	.09
• Female	4.29 (.99)	5.17 (1.72)	.18
- High Density Lipoprotein	1.07 (.42)	1.57 (.55)	< .001
• Male	1.00 (.54)	1.33 (.62)	.16
• Female	1.15 (.17)	1.73 (.43)	.001
- Triglycerides	4.02 (1.65)	3.01 (1.71)	.031
• Male	4.60 (1.36)	3.42 (1.35)	.029
• Female	3.24 (1.77)	2.74 (1.88)	.50
- Low Density Lipoprotein	2.35 (.30)	2.14 (.49)	.033
• Male	2.32 (.38)	2.30 (.33)	.87
• Female	2.40 (.14)	2.03 (.56)	.032

PAD (+): diabetes patients with peripheral arterial disease.

PAD (-): diabetes patients without peripheral arterial disease.

*SD*, Standard deviation.

### Components of metabolic syndrome

Table 3 presents the analysis of components of metabolic syndrome as a risk factor for PAD in the diabetic foot. In the univariate analysis, PAD of diabetic foot is significantly associated to the definition of the waist of the IDF and AHA / NHLBI than that of the NCEP-ATP III. No other element of MS is significantly related to PAD. Table 3 presents the multivariate analysis of the elements of MS according to the two definitions of abdominal obesity. In multivariate analysis of the elements of MS of IDF and AHA / NHLBI definitions, those significantly associated with PAD are abdominal obesity and abnormal levels of HDL. The same elements were found in the definition of the NCEP- ATPIII.

**Table 3:** Analysis of MS and its components as risk factors of PAD of diabetic foot

	**PAD (+)**	**PAD (-)**	***p***	**Crude OR**
	**n = 19**	**n = 48**		**(95% CI)**
***Elements of Metabolic Syndrome, n (%)***				
Abnormal waist circumference^*^	16 (84.2)	18 (37.5)	.001	8.9 (2.3; 34.8)
Abnormal waist circumference^§^	11 (57.9)	16 (33.3)	.06	2.8 (0.9; 8.2)
High density lipoprotein-Cholesterol male < 1.03 or female < 1.29 mmol/L	10 (52.6)	13 (27.1)	.047	3.0 (1.0; 9.0)
Triglycerides?≥?1.7 mmol/L	9 (47.4)	16 (33.3)	.25	1.8 (0.6; 5.3)
Blood pressure ≥130/85 mmHg or Hypertension on treatment	10 (52.6)	25 (52.1)	.97	1.0 (0.4; 3.0)
***Metabolic Syndrome n, (%)***				
IDF criteria	14 (73.7)	18 (37.5)	.008	4.7 (1.4; 15.1)
NCEP-ATPIII criteria	13 (68.4)	26 (54.2)	.29	1.8 (0.6; 5.6)
AHA/NHLBI criteria	16 (84.2)	23 (47.9)	.007	5.8 (1.5; 22.5)

^*^≥?94 cm in men, ≥ 80 cm.

^§^≥?102 cm in men, ≥ 88 cm in women.

PAD (+): patients with peripheral arterial disease.

PAD (-): patients without peripheral arterial disease.

### Metabolic syndrome

Table 4 presents the analysis of different definitions of the metabolic syndrome as a risk factor in PAD of diabetic foot. We had a significant association of definitions of the IDF and AHA / NHLBI with a better association with the criteria of the AHA / NHLBI.

**Table 4:** Logistic multivariate analysis of elements of the metabolic syndrome according to different definitions

	With waist circumference defined as IDF and AHA/NHBLI^*^		**With waist circumference defined as NCEP-ATP III**^**§**^	
	***p***	***Adjusted OR***	***p***	***Adjusted OR***
Abnormal waist circumference^*^	.002	13.8 (2.6; 73.8)	-	-
Abnormal waist circumference^§^	-	-	.020	5.3 (1.3; 21.7)
High density lipoprotein-Cholesterol male < 1.03 or female < 1.29 mmol/L	.013	6.5 (1.5; 28.2)	.006	8.2 (1.8; 36.8)
Triglycerides?≥?1.7 mmol/L	.58	1.5 (0.4; 6.5)	.15	2.6 (0.7; 10.0)
Blood pressure ≥130/85 mmHg or Hypertension on treatment	.99	1.0 (0.2; 4.1)	.38	1.8 (0.5; 6.4)

^*^≥?94 cm in men, ≥ 80 cm.

^§^≥?102 cm in men, ≥ 88 cm in women.

## Discussion

In this study, we found that better correlation of PAD of diabetic foot disease was with the definition of AHA / NHLBI. Limitation of study was due to that patients with arterial hypertension were in treatment. This is a cross-sectional study and we not able to measure the direct effect of risks factors.

No studies found in the literature have been performed in the patient population with a diabetic foot. Several studies found an association of PAD with MS according to the WHO criteria [19,20] unlike the study by Wong et al. [21]. Concerning the association of PAD with MS according to NCEP-ATPIII, several studies found an association and others did not [19,22-24]. The study by Larsson et al. goes in the direction of ours, especially since it is cohort study [19]. The risk of having PAD in another study was higher with the five elements of the MS combined [22], a study in Spain showed a 14 times higher risk of having a PAD in the presence of MS [23]. On the criteria of the AHA / NHLBI, one study found no association with PAD [25]. The difference with our study is that the analysis is done with a logistic regression in combination of other factors. On the IDF criteria, a study found no relationship with PAD in a population of patients aged 50 to 75 years, without specification of diabetes status [22]. The same results were found in a population of Indian patients [24]. A study showed a better association for IDF criteria than for the AHA / NHLBI criteria, in contrast to our study [26]. The association between MS and PAD in the diabetic foot is more significant for the definition of AHA / NHLBI than that of the IDF in our study. No association was found with the definition of the NCEP-ATPIII. Thus, in our study, the risk is higher using the definition of the AHA / NHLBI. The difference between the criteria of AHA / NHLBI and of the NCEP-ATPIII is situated at the android obesity. Waist circumference to consider is?≥?94 cm in men and?≥?88 cm in women for the diagnosis of abdominal obesity in sub-Saharan Africans. The difference between the criteria of AHA / NHLBI and the IDF is at the level of abdominal obesity which is an obligatory criterion for the IDF. With a higher risk with the criteria of the AHA / NHLBI compared to the IDF (5.8 against 4.7), so it is important not to consider abdominal obesity as obligatory criteria for assessing the risk of developing PAD in the presence of MS like JIS definition [3].

Regarding the elements of MS, in univariate analysis in our study, the definition of abdominal obesity according to IDF and AHA / NHLBI is associated with PAD in diabetic foot contrary to the definition of the NCEP-ATPIII. In addition, in the multivariate analysis with other elements of the MS, the two definitions of abdominal obesity are associated with MS. The correlation is still higher than with the IDF and AHA / NHLBI. It spends 13.8 with the definition of IDF and AHA / NHLBI to 5.3 with that of the NCEP-ATPIII. This further demonstrates that the definition of abdominal obesity adopted in the diabetic population of sub-Saharan Africa as a risk factor for PAD in the diabetic foot should be that of the IDF and AHA / NHLBI and not that of the NCEP-ATPIII. The fact that it is only in the presence of other elements of the MS that abdominal obesity as defined by the NCEP-ATPIII become a risk factor for further demonstrates the weakness of the limit imposed for the significance of waist circumference in the definition of abdominal obesity. The risk of having PAD in the diabetic foot when the patient has abdominal obesity according to the criteria of the IDF and AHA / NHLBI increases in the presence of other elements of the MS as it goes from 8.9 to 13.8. This means that the association of this definition of abdominal obesity with other elements of MS, especially an abnormal rate of HDL increases the risk of having PAD. The abnormal level of HDL is the second factor involved in PAD diabetic foot after abdominal obesity. The risk rose from 3.0 in the univariate analysis at 6.5 with the criteria of the IDF and AHA / NHLBI to 8.5 with the criteria of NCEP-ATPIII in the multivariate analysis. Be it single or in the presence of other components of MS, an abnormal HDL fact evokes the presence of PAD in diabetic foot in sub-Saharan Africa but with a higher risk in the presence of other components of MS. The presence of the other two elements of MS, hypertension and abnormal levels of TG, do not imply the presence of PAD in the diabetic foot in our study, both in univariate or multivariate analysis. Relying on these data, it is only by integrating them into the diagnostic criteria for MS that they are considered.

Our study has some limitations that are important to report. It was a case–control study; the direct risk has not been evaluated. In addition, the study was conducted in a reference center that is a tertiary institution. Reflected in the structure of primary health care cannot be assessed, but the results can be compared to those made in other countries of sub-Saharan Africa, given the similarity of the study sites.

## Conclusion

The definition of MS which better evaluates the risk to have a PAD in diabetic foot is the AHA / NHLBI definition. Thus, abdominal obesity should be defined according to the recommendations of the IDF and AHA / NHLBI and should not be an obligatory criterion in the definition of MS for research risk to have PAD in diabetic foot disease on sub-Saharan Africa.

## Abbreviations

ABI: Ankle brachial index; AHA/NHLBI: American Heart Association / National Heart, Lung and Blood Institute; CI: Confidence interval; HDL: High density lipoprotein; IDF: International Diabetes Federation; JIS: Joint interim statement; LDL: Low density lipoprotein; MS: Metabolic syndrome; NCEP-ATPIII: National Cholesterol Education Program-Adult Treatment Panel III; OR: Odds ratio; PAD: Peripheral arterial disease; TC: Total cholesterol; TG: Triglycerides

## Competing interests

The authors declare that they have no competing interests.

## Authors’ contributions

JJRB conceived and designed the study, collected, analyzed and interpreted data, and wrote the manuscript. JB, ES and JCM conceived and designed the study, and performed a critical analysis of the manuscript. All authors approved the final version of the manuscript. All authors agreed to be accountable for all aspects of the work in ensuring that questions related to the accuracy or integrity of any part of the work are appropriately investigated and resolved.

## References

[B1] International Diabetes Federation IDF diabetes atlas 5th edition [http://www.idf.org/sites/default/files/5E_IDFAtlasPoster_2012_FR.pdf]

[B2] International Diabetes Federation The IDF consensus worldwide definition of the metabolic syndrome [http://www.idf.org/webdata/docs/IDF_Meta_def_final.pdf]

[B3] Alberti KGMM Eckel RH Grundy SM Zimmet PZ Cleeman JI Donato KA Fruchart JC James WPT Loria CM Smith SC Harmonizing the metabolic syndrome: a joint interim statement of the international diabetes federation task force on epidemiology and prevention; national heart, lung, and blood institute; american heart association; world heart federation; international atherosclerosis society; and international association for the study of obesity Circulation 2009 120 1640 1645 10.1161/CIRCULATIONAHA.109.192644 19805654

[B4] Alberti KG Zimmet P Shaw J The metabolic syndrome–a new worldwide definition Lancet 2005 366 1059 1062 10.1016/S0140-6736(05)67402-8 16182882

[B5] National Heart Lung and Blood Institute Third Report of the National Cholesterol Education Program (NCEP) Expert Panel on Detection, Evaluation, and Treatment of High Blood Cholesterol in Adults (Adult Treatment Panel III): Final Report [http://www.nhlbi.nih.gov/guidelines/cholesterol/atp3full.pdf] 12485966

[B6] National Heart Lung and Blood Institute Third Report of the Expert Panel on Detection, Evaluation, and Treatment of the High Blood Cholesterol in Adults (Adult Treatment Panel III): Executive Summary [http://www.nhlbi.nih.gov/guidelines/cholesterol/atp3xsum.pdf]

[B7] Grundy SM Brewer HB Jr Cleeman JI Smith SC Jr Lenfant C Definition of metabolic syndrome: report of the national heart, lung, and blood institute/american heart association conference on scientific issues related to definition Circulation 2004 109 433 438 10.1161/01.CIR.0000111245.75752.C6 14744958

[B8] Tchakonté B Ndip A Aubry P Malvy D Mbanya JC The diabetic foot in Cameroon Bull Soc Pathol Exot 2005 98 2 94 98 16050373

[B9] Erasmus RT Soita DJ Hassan MS Blanco-Blanco E Vergotine Z Kengne AP Matsha TE High prevalence of diabetes mellitus and metabolic syndrome in a South African coloured population: baseline data of a study in Bellville, Cape Town S Afr Med J 2012 102 11 Pt 1 841 844 23116739 10.7196/samj.5670

[B10] Kengne AP Limen SN Sobngwi E Djouogo CFT Nouedoui C Metabolic syndrome in type 2 diabetes: comparative prevalence according to two sets of diagnostic criteria in sub-Saharan Africans Diabetol Metab Syndr 2012 4 22 10.1186/1758-5996-4-22 22650602 PMC3407752

[B11] Abbas ZG Archibald LK Epidemiology of the diabetic foot in Africa Med Sci Monit 2005 11 8 262 270 16049394

[B12] World Health Organization Waist circumference and waist–Hip ratio: report of a WHO expert consultation [http://whqlibdoc.who.int/publications/2011/9789241501491_eng.pdf]

[B13] American Diabetes Association Peripheral arterial disease in people with diabetes Diabetes Care 2003 26 3333 3341 14633825 10.2337/diacare.26.12.3333

[B14] Potier L Abi Khalil C Mohammedi K Roussel R Use and utility of ankle brachial index in patients with diabetes Eur J Vasc Endovasc Surg 2011 41 1 110 116 10.1016/j.ejvs.2010.09.020 21095144

[B15] Premalatha G Ravikumar R Sanjay R Deepa R Mohan V Comparison of colour duplex ultrasound and ankle-brachial pressure index measurements in peripheral vascular disease in type 2 diabetic patients with foot infections J Assoc Physicians India 2002 50 1240 1244 12568206

[B16] ATP III at-a-glance: quick desk reference [http://www.nhlbi.nih.gov/guidelines/cholesterol/atglance.htm]

[B17] Abramson JH WINPEPI updated computer programs for epidemiologists, and their teaching potential Epidemiol Perspect Innov 2011 8 1 10.1186/1742-5573-8-1 21288353 PMC3041648

[B18] Kengne AP Amoah AG Mbanya JC Cardiovascular complications of diabetes mellitus in sub-Saharan Africa Circulation 2005 112 23 3592 3601 10.1161/CIRCULATIONAHA.105.544312 16330701

[B19] Larsson I Lindroos A Lystig TC Näslund I Sjöström L Three definitions of the metabolic syndrome: relations to mortality and atherosclerotic morbidity Metab Syndr Relat Disord 2005 3 2 102 112 10.1089/met.2005.3.102 18370717

[B20] Wang J Ruotsalainen S Moilanen L Lepistö P Laakso M Kuusisto J Metabolic syndrome and incident end-stage peripheral vascular disease: a 14-year follow-up study in elderly Finns Diabetes Care 2007 30 12 3099 3104 10.2337/dc07-0985 17848614

[B21] Wong J Molyneaux L Constantino MI Twigg SM Yue DK The metabolic syndrome in type 2 diabetes: When does it matter? Diabetes Obes Metab 2006 8 6 690 697 10.1111/j.1463-1326.2005.00565.x 17026494

[B22] López-Suárez A Beltrán-Robles M Elvira-González J Alwakil M Bascuñana-Quirell A Rosal-Obrador J Badani-Gutiérrez H Oliver-Pece M Pons-Raga A Ruiz-DeCastroviejo J Does diagnosis of metabolic syndrome predict the likelihood of peripheral arterial disease as defined by a low ankle-brachial index? Eur J Cardiovasc Prev Rehabil 2008 15 6 693 697 10.1097/HJR.0b013e32830c1cc5 18756176

[B23] Schmolling Y del Valle FJ Pérez de Oteyza C de Lucas A Brasero F Fajardo F Ankle-brachial index testing is particularly indicated in patients with metabolic syndrome but without known arterial disease Rev Clin Esp 2008 208 4 175 181 10.1157/13117038 18381001

[B24] Jaquet A Deloumeaux J Dumoulin M Bangou J Donnet J-P Foucan L Metabolic syndrome and Framingham risk score for prediction of cardiovascular events in Caribbean Indian patients with blood glucose abnormalities Diabetes Metab 2007 34 2 177 181 18353700 10.1016/j.diabet.2007.10.005

[B25] Papa G Degano C Iurato MP Licciardello C Maiorana R Finocchiaro C Macrovascular complication phenotypes in type 2 diabetic patients Cardiovasc Diabetol 2013 12 20 10.1186/1475-2840-12-20 23331854 PMC3558439

[B26] Brevetti G Laurenzano E Giugliano G Lanero S Brevetti L Luciano R Chiariello M Metabolic syndrome and cardiovascular risk prediction in peripheral arterial disease Nutr Metab Cardiovasc Dis 2010 20 9 676 682 10.1016/j.numecd.2009.05.016 19699069

